# Disseminated Gonococcal Infection Complicated by Prosthetic Joint Infection: Case Report and Genomic and Phylogenetic Analysis

**DOI:** 10.1093/ofid/ofaa632

**Published:** 2020-12-18

**Authors:** Osakpolor Ogbebor, Tatum D Mortimer, Kyra Fryling, Jessica J Zhang, Nitin Bhanot, Yonatan H Grad

**Affiliations:** 1 Division of Infectious Diseases, Allegheny General Hospital, Allegheny Health Network, Pittsburgh, Pennsylvania, USA; 2 Department of Immunology and Infectious Diseases, Harvard T.H. Chan School of Public Health, Boston, Massachusetts, USA

**Keywords:** antibiotic resistance, disseminated gonococcal infection, genomics, *Neisseria gonorrhoeae*, prosthetic joint infection

## Abstract

*Neisseria gonorrhoeae* infections have been increasing globally, with prevalence rising across age groups. In this study, we report a case of disseminated gonococcal infection (DGI) involving a prosthetic joint, and we use whole-genome sequencing to characterize resistance genes, putative virulence factors, and the phylogenetic lineage of the infecting isolate. We review the literature on sequence-based prediction of antibiotic resistance and factors that contribute to risk for DGI. We argue for routine sequencing and reporting of invasive gonococcal infections to aid in determining whether an invasive gonococcal infection is sporadic or part of an outbreak and to accelerate understanding of the genetic features of *N gonorrhoeae* that contribute to pathogenesis.

Since its nadir in 2009, the rate of *Neisseria gonorrhoeae* infections in the United States has increased across age groups [1]. This rise has coincided with joint replacements taking place at younger ages. In this study, we report a case of disseminated gonococcal infection (DGI) from Pittsburgh, Pennsylvania in which these 2 trends intersect: an individual with DGI complicated by prosthetic joint infections (PJIs). We also report the genome sequence of the isolate, including characterization of resistance genes, putative virulence factors, and the phylogenetic lineage. We review the literature, describing the following: (1) advances in sequence-based prediction of antibiotic resistance in *N. gonorrhoeae*; (2) pathogen factors known or proposed to influence the likelihood of invasive gonococcal infection; and (3) the use of sequencing and phylogenetics to aid in understanding whether DGI cases represent sporadic instances or part of an outbreak cluster—an issue particularly relevant in this case, given a recent outbreak of DGI in Michigan [2]. Routine sequencing of invasive isolates will aid in efforts to define the pathogen genetic risk factors for invasive disease as well as improve surveillance and guide public health interventions.

## CASE REPORT

In fall 2019, a 59-year-old white man with a history of left knee replacement 8 years ago presented to the emergency department of a hospital in Pennsylvania with a 2-day history of pain and swelling involving multiple joints. He first noticed pain, swelling, and redness over the dorsum of his left hand, followed by similar symptoms in the right wrist, shoulders, right hip, and left knee. The joint pain was exacerbated by movement and limited weight-bearing. He reported no pain on micturition or penile discharge. He reported he was monogamous. On exam, he appeared uncomfortable. His temperature was 36.9°C. Joint exam revealed tenderness in his shoulders and left 2nd to 5th metacarpophalangeal joints, swelling and erythema of the dorsum of the left hand, and decreased range of movement in his shoulders, knees, and right hip. Laboratory work revealed white blood cell count (WBC) of 15.9 × 10^3^ cells/mL, with 89% neutrophils. The erythrocyte sedimentation rate was greater than 130 mm/hr and C-reactive protein was 33 mg/dL. Human immunodeficiency virus and rapid plasma reagin were negative. Arthrocentesis of the left knee yielded purulent fluid, with a WBC of 162 000 cells/mL, 89% neutrophils, and red blood cell count of 5000 cells/mL.

The differential diagnosis included joint infection due to commonly encountered pathogens, such as staphylococci, DGI, and migratory polyarticular arthritis secondary to a rheumatological disease. He was started empirically on vancomycin and cefepime, pending culture results, and changed to vancomycin and ceftriaxone after infectious disease consult. After culture from the knee aspirate fluid grew *N. gonorrhoeae*, he acknowledged multiple recent unprotected heterosexual encounters.

E-test of the *N. gonorrhoeae* isolate showed an azithromycin minimum inhibitory concentration (MIC) of 0.38 µg/mL, ceftriaxone MIC of <0.016 µg/mL, and ciprofloxacin MIC of 0.003 µg/mL. Management included irrigation and debridement of the left knee with synovectomy and polyethylene liner exchange, arthroscopic irrigation and debridement of the right hip, as well as 4 weeks of intravenous ceftriaxone and a single 1-gram dose of oral azithromycin. He then received 300 mg of cefdinir twice a day for four and a half months, given concern for PJI. His recovery was complicated by tenosynovitis of the left 2nd and 4th compartment with a torn extensor pollicis longus tendon, requiring tendon transfer and repair. He has had no reported recurrence of symptoms.

The *N. gonorrhoeae* isolate, which appeared piliated and opaque on culture, underwent genome sequencing on the Illumina platform and analysis for resistance mutations and loci postulated to be associated with invasiveness [3–6] (see Supplemental Material). Analysis confirmed the absence of known antibiotic resistance determinants for ceftriaxone, with no known resistance variants in *penA*, *rpoB*, or *rpoD* [3, 6]. Likewise, no variants in *gyrA* or *parC* that confer resistance to ciprofloxacin were observed [3]. The isolate’s mosaic *mtrD* with a K823E mutation and the A39T mutation in *mtrR* can increase the azithromycin MIC by altering the MtrCDE efflux pump and increasing its expression, but do not on their own increase the MIC above clinical thresholds for resistance [3, 4]. No other known azithromycin resistance conferring mutation was observed, with no resistance variants in the 23S rRNA or *rplD* genes [5].

Although no genetic basis for dissemination or invasive disease in *N. gonorrhoeae* has been well established, several genetic loci have been speculated to be involved in invasiveness and serum resistance, including the gonococcal genetic island [7], the *opa* genes [8], the genes *porB* [9] and *lptA* [10], and the *lgt* operon [11, 12]. The isolate causing this case lacked the gonococcal genetic island and encoded wild-type *lptA* and *porB1b*. The *lgt* operon codes for glycosyl transferases that mediate biosynthesis of lipooligosaccharide (LOS) and several of the genes in this locus are phase variable, thus influencing the nature of the LOS [13]. Assessment of phase variation indicated that *lgtA* and *lgtC* were off and *lgtD* was on; however, the in vitro passaging of the clinical isolate may have resulted in genetic changes in these loci, rendering interpretation of the phase variation at these loci unclear. The 11 *opa* loci in the genome each have 2 hypervariable regions and could not be resolved and typed by the short sequencing reads.

**Figure 1.**
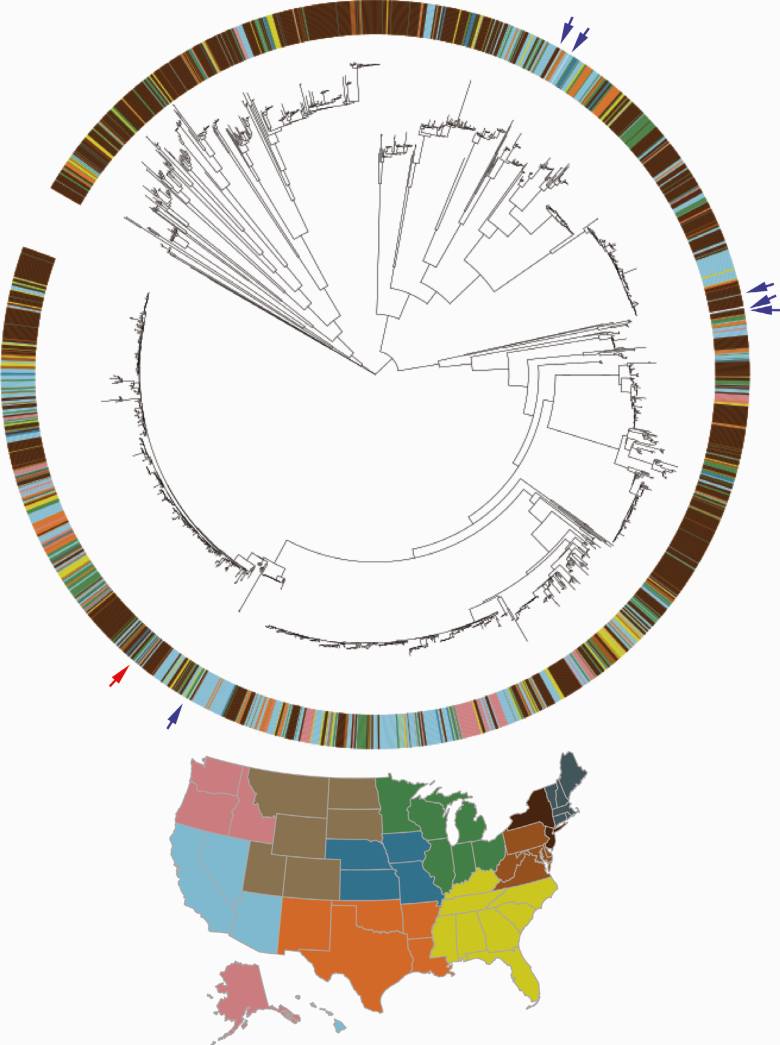
Isolates from disseminated infections are present in multiple gonococcal lineages. To put the isolate from this case in context (denoted by a red arrow) with currently circulating strains in the United States and those from other recently reported disseminated infections, we assembled a collection of publicly available whole-genome sequencing data from US isolates collected since 2010 (2070 isolates, all reported from uncomplicated gonococcal infections) and 6 isolates from disseminated gonococcal infections reported in a recent genomic epidemiology study in Australia [14], and we generated a midpoint rooted, maximum likelihood phylogeny with predicted recombinations removed. The isolates from Australian joint infections are marked with blue arrows. The colors in the annotation ring indicates the geographic location of isolation, as indicated by the map of US Health and Human Services regions.

Phylogenetic analysis revealed that the clinical isolate is derived from an internationally circulating lineage of antibiotic-susceptible *N. gonorrhoeae* with NG-MAST 20638 and MLST 11428. No other isolate causing DGI has been reported from this lineage to date. Comparison with recently reported sporadic cases of DGI in a large genomic epidemiology study [14] revealed that the isolates causing those infections derive from distinct genetic lineages (see Figure 1).

### Patient Consent Statement

Patient consent was obtained. Institutional Review Board review is not required for this activity, for, as a case report, this work neither produces generalizable knowledge nor is it an investigation of a US Food and Drug Administration-regulated product.

## DISCUSSION

Disseminated gonococcal infection can present with dermatitis, migratory arthritis, and tenosynovitis [15]. In this case, the patient presented with migratory arthritis and left-hand tenosynovitis. This case of DGI was complicated by PJI, with only one other case to our knowledge reported in the literature [16], and DGI-associated tendon rupture.

The rarity of gonococcal PJI may be due to DGI as an uncommon manifestation of gonorrhea and the low prevalence of joint replacements in the age group of patients at highest risk of gonococcal infection [15, 17]. However, these groups are increasingly overlapping: more people are having prosthetic joint surgeries at earlier ages [17], and rates of gonococcal infections in the United States rose 63% from 2014 to 2018, with cases of gonorrhea in individuals aged 40+ years more than doubling [1].

The recommended treatment for uncomplicated gonococcal infection is single-dose intramuscular ceftriaxone plus single-dose oral azithromycin, and recommendations for DGI are intravenous antibiotics for 24–48 hours, transitioning on clinical improvement to oral agents, with a total treatment duration of 1 week [18]. For PJI caused by organisms other than *Staphylococci*, Infectious Diseases Society of America (IDSA) guidelines recommend 4 to 6 weeks of pathogen-specific intravenous or highly bioavailable oral antimicrobial therapy after debridement and retention of the prosthesis [19]. In the absence of specific recommendations for *N. gonorrhoeae* PJI, this patient received a single dose of oral azithromycin and a 4-week course of ceftriaxone.

Treatment for gonorrhea is most often empiric, because resistance testing is rarely performed. However, the rising rate of antibiotic resistance highlights the importance of rapid diagnostics to inform clinical treatment regimens [20, 21]. Advances in deoxyribonucleic acid sequence-based antibiotic resistance prediction make this a promising direction. Polymerase chain reaction-based tests for ciprofloxacin resistance [22] and growing understanding of the genetic basis of resistance to ceftriaxone and azithromycin provide an evidence base for development of genotypic assays for predicting phenotypic resistance to these antibiotics [23, 24]. In the case presented here, the genotype predicted susceptibility to ciprofloxacin, ceftriaxone, and azithromycin, in keeping with the observed phenotype. Beyond the categorical determination of susceptibility, the variants observed in *mtrD* and *mtrR*, each expected to slightly increase the azithromycin MIC, were consistent with the Etest result. The accuracy of quantitative MIC prediction suggests that advances in genotype-to-phenotype models may make these assays useful for nuanced clinical decision making.

The factors that influence the likelihood of invasive gonococcal disease remain incompletely understood. Host factors predisposing to disseminated disease include innate or acquired complement deficiency [25, 26]. The pathogen factors contributing to invasiveness have been more challenging to establish, because they have been based on small numbers of cases. An outbreak of DGI in the late 1970s and early 1980s was caused by an auxotype requiring arginine, hypoxanthine, and uracil (the AHU auxotype) [27–31], raising suspicion that this auxotype-defining lineage carried genetic determinants that promoted invasiveness. However, this auxotype seems to have stopped circulating, and further genomic and phenotypic characterization of this lineage remains to be done. Other genetic loci speculated to be virulence factors include the gonococcal genetic island [7], the *opa* genes [8], the genes *porB* [9] and *lptA* [10], and the *lgt* operon [11, 12], with several of these loci contributing to escape of complement-mediated killing [32]. Recent outbreaks, including one among heterosexuals in Michigan, have renewed questions about the genetic predisposition of particular lineages to invasiveness [2, 33].

Routine reporting of genome sequences of invasive *N. gonorrhoeae* isolates together with the clinical contexts can aid in the effort to define the genetic basis for gonococcal virulence and represent an important complement to in vitro and animal-model studies. For comparison, recent studies using sequence data from isolates collected over many years and countries has expanded our knowledge of the genetic modulators of antimicrobial resistance in clinical isolates of *N. gonorrhoeae* as well as its adaptation to anatomical sites of infection [4–6, 34, 35]. Likewise, the genome sequences of *Neisseria meningitidis* isolates causing sporadic cases and outbreaks of urethritis have aided in understanding the genetic basis of meningococcal adaptation to the urogenital niche and in clarifying the importance of putative virulence loci [34, 36–38].

Although *N. gonorrhoeae* genome sequencing has primarily relied on cultured specimens, recent advances demonstrated the potential of sequencing directly from patient specimens [39, 40]. The most likely near-term use of these technologies includes point-of-care prediction of antibiotic susceptibility based on genome sequence either by direct assessment of one or more loci [23, 39, 40] or by phylogenetic-neighbor typing methods [41]. The use of long-read platforms to sequence directly from patient specimens also contributes to efforts to identify loci that contribute to invasiveness. This approach will allow for querying phase-variable sites (such as in the *lgt* operon) directly, thereby eschewing the confounding phase alterations that may arise during in vitro passage. In addition, long-read sequencing will allow for the comprehensive characterization of the Opa repertoire that is challenging to do with short-read sequencing platforms.

## CONCLUSIONS

Reporting of sporadic and outbreaks of DGI cases together with genome sequence data and host risk factors can inform and enable similar efforts to combine data and uncover the *N. gonorrhoeae* genetic contributors to invasiveness as well as understand the extent to which cases reflect gonococcal lineages with a higher risk of invasion. At least 10 individual cases of DGI and one DGI outbreak have been published and indexed on PubMed over the past year [2, 42–52]. Sequences from these isolates would address several key questions. First, to what extent are DGI cases sporadic, and to what extent do they represent a lineage’s propensity to cause invasive disease? For example, of the isolates from DGI cases in Australia [14] included in the phylogeny, some isolates are clustered and others appear sporadic (Figure 1). Adding more isolate genomes to this phylogeny will help assess whether the clustered cases represent outbreaks, and additional outbreak lineages will provide statistical power to identify the genetic basis for invasiveness. Second, although disseminated infection remains rare and rates appear to have declined over the past several decades [53], is the lower rate attributable to changes in circulating strains? Third, if there is a DGI cluster, how geographically and demographically widespread is it? For example, the reporting of genome sequences from the recent Michigan DGI outbreak will inform on whether the case reported here reflects spread of an invasive lineage and inform efforts for surveillance.

The number of genome sequences from clinical *N. gonorrhoeae* isolates in public databases is over 13 000 and steadily growing. Just as the subset of these isolates for which antibiotic resistance data has helped expand our understanding of the genetic basis of resistance [4, 5, 34], routinely sequencing and reporting invasive strains will increase the statistical power to address critical questions of *N. gonorrhoeae* virulence and help inform public health surveillance and interventions.

## Supplementary Data

Supplementary materials are available at Open Forum Infectious Diseases online. Consisting of data provided by the authors to benefit the reader, the posted materials are not copyedited and are the sole responsibility of the authors, so questions or comments should be addressed to the corresponding author.

ofaa632_suppl_Supplementary_Table_1
